# Clinical and Laboratory Risk Factors of Early Poor Outcome in Patients With Childhood‐Onset Lupus Nephritis—A Single‐Center Retrospective Study

**DOI:** 10.1002/iid3.70146

**Published:** 2025-02-11

**Authors:** Wei Qijiao, Huang Fujia, Yang Bing, Wang Changyan, Zhong Linqing, Dong Yanqing, Wang Wei, Song Hongmei

**Affiliations:** ^1^ Department of Pediatrics, Peking Union Medical College Hospital, Peking Union Medical College Chinese Academy of Medical Sciences Beijing China; ^2^ Department of Rheumatology Children's Hospital of Fudan University Shanghai China; ^3^ Department of Pediatrics Baise People's Hospital Baise China; ^4^ Neonatal Intensive Care Unit, Department of Pediatrics, Peking Union Medical College Hospital, Peking Union Medical College Chinese Academy of Medical Sciences Beijing China

**Keywords:** children, early prognosis analysis, lupus nephritis, risk factors

## Abstract

**Objective:**

*Childhood‐onset* lupus nephritis (LN) tends to be more severe than in adults. A significant correlation between remission at 3 months of induction therapy and remission after 3 years was found in adults. While few studies on the risk factors of poor early prognosis in children with LN were made. Thus, this study investigated the risk factors of early poor response to help doctors develop effective treatment strategies.

**Methods:**

A total of 99 LN children at Peking Union Medical College Hospital from January 2012 to January 2018 were evaluated and clinical data were retrospectively collected. In the study, a complete remission (CR) was defined as laboratory test results were completely normal, including blood routine, renal function, albumin, complement, and erythrocyte sedimentation rate, and the 24‐h urinary total protein (24 h UTP) was less than 150 mg. After 3 months of treatment, 15 children achieved CR, and they were in good prognosis group (*n* = 15). While 84 did not achieve CR, and they were in poor prognosis group (*n* = 84). We compared the differences of clinical and laboratory indicators between the two groups.

**Results:**

According to inclusion and exclusion criteria, 99 of 116 children with LN were included in this study. And 15 LN children were in good prognosis group. While 84 patients were in poor prognosis group. The incidence of rash (32.1% vs. 6.7%, *p* = 0.036) and oral ulcer (81.0% vs. 53.3%, *p* = 0.027) in poor prognosis group is higher than that in the good prognosis group. The 24 h UTP (g) [2.46 (1.41, 4.86) vs. 0.56 (0.30, 0.66), *p* < 0.001] and the serum creatinine (umol/L) [53.0 (40.3, 65.0) vs. 39.0 (29.8, 51.5), *p* = 0.017] were higher in poor prognosis group. The albumin (g/L) (28.7 ± 8.1 vs. 34.5 ± 5.3, *p* = 0.003) is lower in poor prognosis group. Logistic regression analysis showed that rash (*p* = 0.036), oral ulcer (*p* = 0.027), high 24 h UTP (*p* < 0.001), high creatinine (*p* = 0.017), and low serum albumin (*p* = 0.003) were significantly associated with poor early prognosis in childhood‐onset LN.

**Conclusion:**

The occurrence of rash cannot be ignored, especially for children with oral ulcers, a comprehensive evaluation of each system should be carried out, so as not to cause inactive treatment and affect the prognosis. High 24 h UTP have a positive predictive value for the early poor outcome of childhood‐onset LN. Active control of proteinuria and achieving rapid renal remission is crucial for good prognosis.

## Summary

- The risk factors of poor early prognosis with LN were first explored in children.
- Three months is a time point to conduct group comparisons aiming to identify adverse prognostic factors earlier and provide guidance for clinicians.
- The occurrence of rash and oral ulcers in the poor prognosis group was significantly higher.
- 24 h UTP quantification and serum creatinine were significantly higher and serum albumin was lower in poor prognosis group.
- Increased 24 h UTP quantification is an independent risk factor for poor prognosis.

## 1 Introduction

Systemic lupus erythematosus (SLE) is an autoimmune connective tissue disease marked by immune‐mediated inflammation. In children, SLE tends to be more severe than in adults and frequently affects multiple organs [1]. One study reported that in South Africa, severe disease presentation and poor outcomes among pediatric SLE patients [2]. Lupus nephritis (LN) is one of the leading causes of mortality in children [3]. LN patients differ from non‐LN patients in the age of SLE diagnosis, treatment modalities, and autoantibody profile and have more frequent, severe manifestations of SLE [4]. Renal impairment affects 33%–75% of pediatric patients with SLE, and more than 80% develop LN within the first 2 years after diagnosis [5]. The interplay between immune dysregulation and inflammatory processes is central to the pathogenesis of LN, where aberrant immune responses lead to kidney inflammation and injury. Studies have shown that various factors, such as elevated urinary protein and serum creatinine, sex, and age, anti‐Sm antibody, can be risk factors for poor prognosis in LN, but the conclusions are not uniform. And their research has primarily concentrated on the risk factors associated with the progression of LN in adults [6, 7, 8, 9]. To our knowledge, one study indicated that in Tunisian children with LN, predictive factors for poor outcomes included a baseline creatinine level exceeding 2.26 mg/dL, elevated proteinuria at baseline, the presence of fibrous crescents identified through renal biopsy, thromboembolic complications, infectious complications, and end‐stage renal disease (ESRD) [10]. Hanaoka et al. [11] found a significant correlation between remission at 3 months of induction therapy and remission after 3 years. Patients with pediatric LN who possess high‐risk factors can be identified early by concurrently assessing their baseline characteristics and initial treatment response [12].

While there are very few studies on the risk factors of poor early prognosis in children with LN. Therefore, further studies should be performed to identify the risk factors of early poor outcomes. This study retrospectively analyzed the clinical data and laboratory indicators of children with LN in our hospital and analyzed the risk factors for poor early prognosis aiming to identify adverse prognostic factors earlier and provide guidance for clinicians.

## 2 Materials and Methods

### 2.1 Study Design and Inclusion Criteria

**Figure 1:**
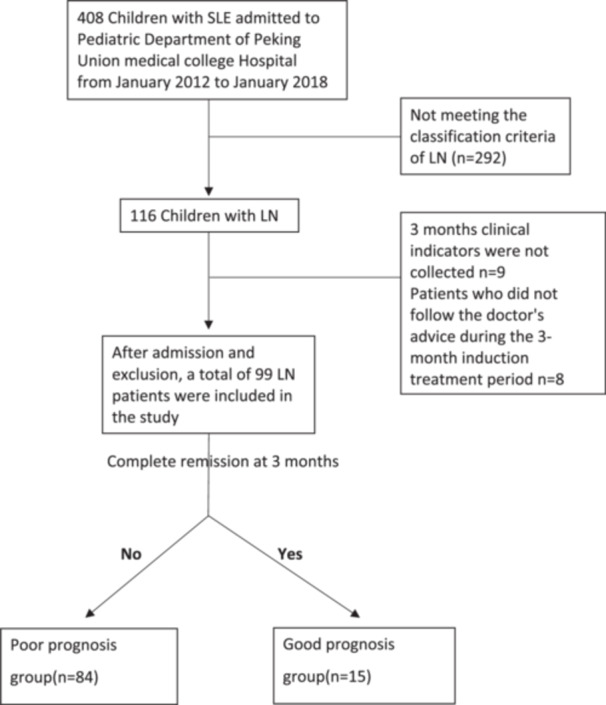
Study flow diagram.

This study was an observational study, and it was approved by the Ethics Committee of Peking Union Medical College Hospital (NO.S‐K885), individual consent was signed by the parents. Four hundred and eight children with SLE admitted to the pediatric department of Peking Union Medical College Hospital from January 2012 to January 2018 were included. Inclusion criteria: (1) Meet the 1997 revised classification criteria of the American College of Rheumatology [13] and the 2012 Systemic Lupus International Collaborating Clinics classification criteria [14] for both SLE and LN. The 2003 International Society of Nephrology/Renal Pathology Society (ISN/RPS) classification [15] was used as a reference standard for the pathological classification of childhood‐onset LN in this study. On the basis of being diagnosed with SLE, if the child has any of the following renal involvement symptoms, it can be diagnosed as LN: (a) If the urine protein test meets any of the following criteria: three qualitative urine protein tests positive within 1 week; or 24‐h urinary total protein (24 h UTP) quantification > 150 mg; Or protein/urinary creatinine > 0.2 mg/mg, or urinary microalbumin levels higher than normal three times within a week; (b) Centrifuge urine with more than five red blood cells per high‐power field of view; (c) Abnormal glomerular and/or tubular function; (d) Renal biopsy (hereinafter referred to as renal biopsy) shows abnormalities, consistent with the pathological changes of LN. (2) Age ≤ 18 years old. (3) 3 months of clinical indicators were collected in our hospital. Exclusion criteria: (1) Incomplete medical history data; (2) Patients who did not follow the doctor's advice during the 3‐month induction treatment period. After admission and exclusion, a total of 99 LN patients were included in the study (Figure 1).

### 2.2 Data Collection

The clinical data of children before treatment and 3, 6, and 12 months after treatment were collected through outpatient medical records and inpatient medical records. The clinical data include general conditions such as sex, age of onset, renal pathological type, and affected system, laboratory tests such as blood routine, 24 h UTP quantification, renal function, anti‐ds‐DNA antibody, complement 3 and 4, and erythrocyte sedimentation rate, and Systemic Lupus Erythematosus Disease Activity Index (SLEDAI). Family history of autoimmunity in the first and second‐degree relatives included type 1 diabetes, SLE, thyroid disease, rheumatoid arthritis and connective tissue disease.

### 2.3 Grouping Criteria

Definition of early poor prognosis: after 3 months of induction therapy with glucocorticoid and immunosuppressants, complete remission (CR) has not been achieved. CR was defined as follows: laboratory test results were completely normal, including blood routine, renal function, albumin, complement, and erythrocyte sedimentation rate, and the 24 h UTP was less than 150 mg. Partial response: (1) ≥ 50% reduction in urinary protein (non‐renal disease range) or (2) 50% improvement in serum creatinine and urinary protein/creatinine or (3) improvement in serum creatinine, urinary protein/creatinine < 1.0 and all other laboratory tests improved compared with before treatment. Nonresponse: The above criteria for remission have not been met [16, 17]. According to the above criteria, after 3 months of treatment, 15 of the 99 children achieved CR, and 84 did not. And the assessment of laboratory tests in two groups were also made at 3, 6, and 12 months (Figure 1).

### 2.4 Statistical Analysis

The Shapiro–Wilk test was used to assess the normality of data. The data were presented as mean ± standard deviation (*x* ± *s*) for normally distributed continuous variables, and as median with Q1–Q3 ranges for continuous variables that do not follow a normal distribution. The ratio was calculated using the Chi‐square test or Fisher test. The included children have complete medical history data, and missing data values were deleted. SPSS (version 25.0) was utilized to compare differences between the two groups using either the *t*‐test or Mann–Whitney *U*‐test. Univariate logistic regression was used to analyze the risk factors associated with poor prognosis, while multivariate logistic regression was employed to identify independent risk factors. A *p*‐value of less than 0.05 was considered statistically significant.

## 3 Results

### 3.1 General Clinical Information and Comparison of Laboratory Tests at Onset

According to inclusion and exclusion criteria, 99 of 116 children were included in this study. After 3 months of treatment, 15 children achieved CR, and they were in good prognosis group (*n* = 15). While 84 did not achieve CR and they were in poor prognosis group (*n* = 84). There were no significant differences in demographic characteristics between the two groups. However, the incidence of rash (*p* = 0.036) and oral ulcers (*p* = 0.027) was notably higher in the poor prognosis group, while other clinical characteristics showed no significant differences. At the onset, the 24 h UTP quantification (g) in the poor prognosis group was significantly elevated compared to that in the good prognosis group [2.46 (1.41, 4.86) vs. 0.56 (0.30, 0.66), *p* < 0.001]. Additionally, serum albumin (g/L) were lower in poor prognosis group than that of good prognosis group (28.7 ± 8.1 vs. 34.5 ± 5.3, *p* = 0.003). The serum creatinine (umol/L) of children with poor prognosis group was significantly higher than that of good prognosis group [53.0 (40.3, 65.0) vs. 39.0 (29.8, 51.5), *p* = 0.017]. There was no statistical difference in other laboratory indicators between the two groups (see Tables 1 and 2). There was no statistically significant difference in renal pathological types between the two groups. Among the 84 and 15 LN children in two groups, 55 (65.5%) and 8 (53.3%) patients underwent renal biopsy, while the remaining 29 refractory patients and 7 nonrefractory patients without renal biopsy were unable to cooperate due to their young age or had contraindications or their parents were concerned about the risk.

**Table 1:** Comparison results of the general clinical information of the two groups of children.

	Poor prognosis group (*n* = 84)	Good prognosis group (*n* = 15)	*p*‐value
Demographic characteristics			
Age of onset (years)	10.7 ± 2.6	9.7 ± 2.6	0.19
Age at diagnosis (years)	11.3 ± 2.4	10.2 ± 2.7	0.10
Time from onset to diagnosis (years)	0.15 (0.00, 0.60)	0.45 (0.00, 0.58)	0.34
Gender (female/%)	67/79.8%	14/93.3%	0.21
Family history	9 (10.7%)	1 (6.7%)	0.63
Clinical features, *n* (%)			
Rash	27 (32.1%)	1 (6.7%)	0.036
Fever	34 (40.5%)	3 (20.0%)	0.13
Joint pain or arthritis	55 (65.5%)	7 (46.7%)	0.17
Mouth ulcers	68 (81.0%)	8 (53.3%)	0.027
Serositis	45 (53.6%)	11 (73.3%)	0.16
Blood involvement	41 (48.8%)	9 (60.0%)	0.58
Neuropsychiatric involvement	15 (17.9%)	2 (13.3%)	1.00
Cardiopulmonary involvement	13 (15.5%)	4 (26.7%)	0.28
Hypertension	56 (66.7%)	11 (73.3%)	0.61
LN class	55 (65.5%)	8 (53.3%)	0.39
I	1 (1.8%)	1 (12.5%)	0.11
II	2 (3.6%)	1 (12.5%)	0.27
III	8 (14.5%)	2 (25.0%)	0.45
IV	22 (40.0%)	2 (25.0%)	0.41
V	10 (18.2%)	1 (12.5%)	0.69
III + V	7 (12.7%)	0 (0.0%)	0.29
IV + V	5 (9.1%)	1 (12.5%)	0.76

*Note:* Measurement data are expressed as mean ± standard deviation (*x* ± *s*) or median, (quartile) [*M* (Q1, Q3)], count data are expressed as proportion (%). Family history of immune disease in the first and second‐degree relatives included type 1 diabetes, SLE, thyroid disease, rheumatoid arthritis, and connective tissue disease.

Abbreviation: LN, lupus nephritis.

**Table 2:** Comparative results of laboratory tests at onset.

	Poor prognosis group (*n* = 84)	Good prognosis group (*n* = 15)	*p*‐value
White blood cells (×10^9^/L)	6.74 (4.73, 9.47)	4.43 (2.75, 10.58)	0.19
Hemoglobin (g/L)	106.1 ± 23.1	110.5 ± 18.1	0.49
Platelets (×10^9^/L)	202 (107, 289)	191 (156, 370)	0.35
24 h UTP (g)	2.46 (1.41, 4.86)	0.56 (0.30, 0.66)	< 0.001
Serum albumin (g/L)	28.7 ± 8.1	34.5 ± 5.3	0.003
Serum creatinine (umol/L)	53.0 (40.3, 65.0)	39.0 (29.8, 51.5)	0.017
Blood urea nitrogen (mmol/L)	6.52 (4.24, 9.42)	5.49 (3.82, 7.17)	0.12
ESR (mm/h)	37.5 (19.0, 75.0)	34.5 (17.0, 60.0)	0.23
C3 (g/L)	0.28 (0.43, 0.61)	0.32 (0.26, 0.56)	0.17
C4 (g/L)	0.02 (0.05, 0.09)	0.05 (0.03, 0.06)	0.45
Anti‐ds‐DNA antibody	61 (72.6%)	11 (73.3%)	0.95
Anticardiolipin antibodies	16 (19.0%)	2 (13.3%)	0.60
Anti‐β2GP1 antibody	17 (20.0%)	3 (20.0%)	0.98
Lupus anticoagulant	29 (34.5%)	3 (20.0%)	0.27
Anti‐Sm antibody	25 (29.8%)	3 (20.0%)	0.44
Anti‐SSA antibody	32 (38.1%)	6 (40.0%)	0.89
Anti‐SSB antibody	9 (10.7%)	2 (13.3%)	0.77
SLEDAI	20 (16, 23)	16 (13, 25)	0.45

*Note:* Measurement data are expressed as mean ± standard deviation (*x* ± *s*) or median, (quartile) [*M* (Q1, Q3)], count data are expressed as proportion (%).

Abbreviations: C3, Complement 3; C4, Complement 4; ESR, erythrocyte sedimentation rate; SLEDAI, Systemic Lupus Erythematosus Disease Activity Index.

### 3.2 Logistic Regression Analysis

Logistic univariate analysis found that oral ulcers, increased 24 h UTP, and decreased albumin were all risk factors for poor prognosis. Logistic multivariate analysis found that increased 24 h UTP quantification was an independent risk factor for poor prognosis (*p* < 0.001) (Table 3).

**Table 3:** Risk factors for poor early prognosis of LN.

	Univariate analysis (*p*‐value)	Multifactor analysis		
		OR	95% CI	*p*‐value
Rash	0.036	4.11	0.35–48.44	0.26
Mouth ulcers	0.027	2.63	0.45–15.35	0.28
24 h UTP	< 0.001	63.99	3.43–1193.22	< 0.001
Serum albumin	0.003	1.17	0.96–1.42	0.10
Serum creatinine	0.017	1.01	0.96–1.05	0.75

Abbreviations: 95% CI, 95% confidence interval; OR, odds ratio.

### 3.3 The Assessment of Two Groups at 3, 6, and 12 Months

After 3 months of treatment, 15 of 99 children achieved CR. The 24 h UTP (g) in the poor prognosis group was significantly higher than that in the good prognosis group [0.52 (0.21, 2.31) vs. 0.09 (0.08, 0.11), *p* < 0.001]. The serum albumin (g/L) of children with poor prognosis group was lower than that of children with good prognosis group (36.5 ± 6.9 vs. 41.6 ± 3.5, *p* < 0.001). ESR (*p* = 0.023) and SLEDAI (*p* < 0.001) in poor prognosis group were higher. At 6 months, the 24 h UTP, SLEDAI in the poor prognosis group was significantly higher, and the serum albumin was lower. At 6 months, 69 and 14 patients in poor and good prognosis groups, respectively, arrived at our hospital for follow‐up. And the CR rate was 36.2% in the poor prognosis group. At 12 months, the CR rate was 59.1%. The follow‐up data are presented in Table 4. Our cohort's CR rate at 6 months was 47.0%, which is lower than another Chinese cohort's data of 65.2% [18]. At this time point, our partial remission rate was 48.2%, higher than the other Chinese cohort's 19.6%. The complete and partial remission rates at 12 months were 53.5% and 39.4%, respectively, while the other Chinese cohort reported rates of 78.3% and 7.6%. Our cohort had a nonresponse rate of 1.4% at 6 months and 1% at 12 months, whereas the other Chinese cohort had higher nonresponse rates of 15.2% and 14.1% at 6 and 12 months, respectively.

**Table 4:** Twelve months follow‐up data of childhood‐onset LN.

	3 months			6 months			12 months		
	Poor prognosis group (*n* = 84)	Good prognosis group (*n* = 15)	*p*‐value	Poor prognosis group (*n* = 69)	Good prognosis group (*n* = 14)	*p*‐value	Poor prognosis group (*n* = 84)	Good prognosis group (*n* = 15)	*p*‐value
White blood cells (×10^9^/L)	8.69 (6.48, 10.68)	9.27 (5.52, 10.66)	0.83	6.1 (4.49, 8.12)	9.53 (7.51, 9.99)	0.63	5.53 (4.08, 7.21)	6.26 (6.02, 7.79)	0.97
Hemoglobin (g/L)	126.1 ± 18.7	130.5 ± 16.7	0.47	133.4 ± 14.9	132.4 ± 12.76	0.97	127.3 ± 16.1	133.7 ± 9.31	0.79
Platelets (×10^9^/L)	255 (209, 296.5)	237.5 (227.5, 284.8)	0.33	289 (237, 332.5)	284.5 (231.8, 349.3)	0.40	295 (236, 334)	286 (228, 340)	0.84
24 h UTP (g)	0.52 (0.21, 2.31)	0.09 (0.08, 0.11)	< 0.001	0.16 (0.08, 0.42)	0.06 (0.05, 0.08)	< 0.001	0.12 (0.06, 0.18)	0.06 (0.04, 0.06)	0.08
Serum albumin (g/L)	36.5 ± 6.9	41.6 ± 3.5	< 0.001	40.9 ± 4.4	43.8 ± 4.05	< 0.001	42.5 ± 3.6	44.3 ± 2.1	0.13
Serum creatinine (umol/L)	47 (41.3, 56.8)	52 (44, 61.5)	0.67	51.5 (42.3, 55.8)	52 (48.5, 63)	0.41	46 (40, 61)	52.5 (43.8, 60.3)	0.59
Blood urea nitrogen (mmol/L)	4.95 (3.78, 6.36)	4.87 (3.51, 6.38)	0.39	3.53 (3.19, 4.19)	4.69 (3.12, 6.19)	0.81	4.18 (2.61, 5.19)	3.56 (3.04, 4.85)	0.05
ESR (mm/h)	17 (9, 28)	10 (4.3, 23)	0.023	10.5 (7, 22)	10 (6.3, 17.5)	0.29	9 (4, 21)	6.5 (3.8, 9.8)	0.021
C3 (g/L)	0.96 (0.83, 1.11)	1.03 (0.89, 1.23)	0.34	0.92 (0.69, 1.07)	1.05 (0.84, 1.35)	0.29	0.94 (0.54, 1.19)	1.09 (0.89, 1.15)	0.42
C4 (g/L)	0.12 (0.08, 0.19)	0.14 (0.13, 0.19)	0.19	0.14 (0.1, 0.16)	0.18 (0.14, 0.20)	0.17	0.1 (0.08, 0.16)	0.15 (0.14, 0.21)	0.21
Anti‐ds‐DNA antibody	24/60	3/12	0.37	25/44	3/11	0.23	21/45	1/13	0.05
SLEDAI	8 (4, 12)	4 (0, 6)	< 0.001	6 (3.3, 8)	4 (0, 8)	0.034	6 (2, 8)	0 (0, 5)	0.042
Complete remission	0	15	< 0.001	25	14	< 0.001	39	14	< 0.001
Partial remission	73	0	< 0.001	40	0	< 0.001	39	0	< 0.001
Remission	73	15	0.21	65	14	1.00	78	14	1.00
Active disease	11	0	0.21	1	0	1.00	1	0	1.00
Relapse rate	0	0	1.00	3	0	1.00	5	1	1.00
Death	0	0	1.00	0	0	1.00	0	0	1.00

*Note:* Measurement data are expressed as mean ± standard deviation (*x* ± *s*) or median, (quartile) [*M* (Q1, Q3)], count data are expressed as proportion (%).

Abbreviations: C3, Complement 3; C4, Complement 4; ESR, erythrocyte sedimentation rate; SLEDAI, Systemic Lupus Erythematosus Disease Activity Index.

### 3.4 Medication of 3 Months Induction Therapy in Childhood‐Onset LN

According to the guidelines and expert consensus for the diagnosis and treatment of SLE, the overall principle for treating LN in our center is as follows: For Class I and II LN, the preferred induction therapy is a combination of glucocorticoids and mycophenolate mofetil. For Class III, IV, III + V, and IV + V LN, the first choice for induction therapy is a combination of glucocorticoids and cyclophosphamide. For children with suboptimal treatment outcomes, cyclophosphamide and mycophenolate mofetil can be interchanged based on glucocorticoid therapy. If both medications are ineffective, calcineurin inhibitors will be used instead. For Class V LN, the preferred induction therapy is a combination of glucocorticoids and calcineurin inhibitors. We mainly determine the treatment plan of patients with non‐known LN class based on their 24 h UTP. If the 24 h UTP was greater than 30 mg/kg, we treated them according to the treatment plan for type III or IV LN. If it was less than 30 mg/kg, we treated them according to type II LN. We found no statistically significant differences in drug treatment between the two groups. We found in our cohort, one child in the poor prognosis group received rituximab treatment in induction therapy (Table 5). One study found add‐on rituximab was an effective and safe rescue therapy for LN children with life‐/organ‐threatening manifestations or treatment‐resistance [19].

**Table 5:** Medication data of 3 months in childhood‐onset LN.

Induction therapy	Poor prognosis group (*n* = 84)	Good prognosis group (*n* = 15)	*p*‐value
Methylprednisolone pulse therapy	54 (64.2%)	8 (53.3%)	0.56
Cyclophosphamide pulse therapy	66 (78.6%)	8 (53.3%)	0.05
Oral corticosteroid	84 (100.0%)	15 (100.0%)	1.00
Hydroxychloroquine	83 (98.8%)	15 (100.0%)	1.00
Mycophenolate mofetil	11 (13.1%)	5 (33.3%)	0.06
Cyclosporine	6 (7.1%)	1 (6.7%)	1.00
Tacrolimus	1 (1.2%)	0 (0.0%)	1.00
Leflunomide	4 (4.8%)	1 (6.7%)	0.57
Rituximab	1 (1.2%)	0 (0.0%)	1.00
Methotrexate	1 (1.2%)	0 (0.0%)	1.00

## 4 Discussion

SLE in children is more severe than in adults and often involves the kidneys [1]. LN is one of the most common causes of death in children [3]. The early diagnosis of LN and the application of immunosuppressants have significantly improved the prognosis of children with LN. Hanaoka et al. [11] found that the remission at 3 months of induction therapy was significantly related to the remission after 3 years, so CR at 3 months is very important for the prognosis. In this study, failure to achieve CR after 3 months of induction therapy with glucocorticoid and immunosuppressants was used as the criterion for early poor prognosis to study the risk factors for early poor prognosis of LN [20].

The rash of SLE mainly includes butterfly erythema, discoid erythema, and some children may develop bullous rash. Studies have found that children with rashes are more likely to be accompanied by photosensitivity, oral ulcers, and hair loss [21]. This study found that the incidence of rash and oral ulcers in the poor prognosis group was significantly higher than that in the good prognosis group. At the same time, logistic univariate analysis found that oral ulcer was a risk factor for poor prognosis of LN. Studies have reported that skin lesions are positively correlated with the severity of SLE disease and major organ damage [22], and children with younger onset ages are less likely to develop rashes than adults [21]. Facial rashes may be a sign of multisystem damage in the process of SLE [23]. Therefore, in clinical diagnosis and treatment, especially for children, the occurrence of rash cannot be ignored, especially for children with oral ulcers, a comprehensive evaluation of each system should be carried out, so as not to cause inactive treatment and affect the prognosis.

The clinical manifestations of LN are diverse, ranging from asymptomatic hematuria and/or proteinuria to nephrotic syndrome to rapidly progressive nephritis with renal impairment [24, 25]. Clinicians often use proteinuria and renal function as the criteria for judging the prognosis of LN [20]. Consistent with the report of this study, the 24 h UTP quantification and serum creatinine in the poor prognosis group were significantly higher than those in the good prognosis group. The serum albumin of the poor prognosis group was lower than that of the good prognosis group. It is well known that children with LN suffer from hypoalbuminemia due to massive protein loss in the urine. Logistic univariate analysis also found that increased 24 h UTP and decreased albumin were risk factors for poor prognosis. Further Logistic multivariate analysis found that increased 24 h UTP was an independent risk factor for poor prognosis. Proteinuria is an important sign of renal injury. Persistent proteinuria can lead to glomerulosclerosis and tubulointerstitial damage and is an independent risk factor for the progression of renal disease to ESRD [26, 27, 28]. Therefore, active control of proteinuria is crucial for prognosis. The therapeutic objectives for managing LN encompass achieving rapid renal remission, preventing flares, avoiding chronic kidney damage, enhancing both survival and quality of life, and minimizing iatrogenic effects. As we see improvements in short‐term outcomes, it becomes increasingly important to focus on balancing the risks associated with long‐term immunosuppressive therapy [29]. Some studies have shown that age and male are also risk factors for poor prognosis of LN [6, 7, 8]. However, this study did not find positive results for the above factors. The population selected in this study was children and adolescents aged 18 or less, while the population reported in other studies included people of all ages (from children to the elderly), which may be one of the reasons why this study did not get a positive result for age. Several studies have found that male sex may be a factor of poor prognosis in SLE [30]. The reasons why this sex difference leads to differences in clinical manifestations and prognosis are not yet fully elucidated, but studies suggest that the role of sex‐hormone is very important, which can affect the effectiveness of therapeutic drugs and thus affect the prognosis of patients [31, 32]. Most of the children in this study have not yet entered puberty, and the sample size is relatively small, so no positive results were obtained. The current guideline recommends to formulate treatment plans based on different renal pathological types. The renal pathological type is of great significance to the evaluation of the prognosis of children [24]. No positive findings were found. Because the clinical manifestations of LN have a relationship with pathological types [25], clinicians have certain clinical experience in the selection of drugs for children who have not undergone renal biopsy during the diagnosis and treatment process. This is one of the reasons that whether the renal puncture or not did not affect the prognosis of the children in this study. Not all children included in the study underwent renal biopsy, and the follow‐up time was short, which also affected the results.

In our study, we found the differences between our cohort and the other Chinese cohort. Ethnically, different populations contributed little for the difference. We thought that the biggest reason for the difference between the two cohorts is the different definitions of CR, PR, and NR. The CR in our cohort was defined as laboratory test results were completely normal, while CR in the other Chinese cohort was defined as sustained Urine Protein Creatinine Ratio (UPCR) < 0.5 mg/mg on an early morning urine. Thus, the CR rate at 6 months was lower, and PR rate was higher.

In our cohort, one child in poorly prognosed patients used rituximab for induction therapy. One study found that treatment with rituximab resulted in improvements in proteinuria, eGFR and serological parameters, including hemoglobin levels, complement 3 levels and anti‐dsDNA antibodies, compared with baseline [19].

In this study, failure to achieve CR after 3 months of hormone and immunosuppressant induction therapy is regarded as the criterion for early poor prognosis, and it is an important symbol for LN to continue to maintain or adjust immunosuppressive therapy. This study still has the following limitations: (1) This study is a retrospective observational study with a short follow‐up period. (2) Not all children underwent renal biopsy, and the pathological type of renal biopsy may affect the prognosis of children. (3) The number of children is small, and the sample size needs to be further expanded.

## 5 Conclusion

In summary, if LN child have rash, oral ulcers, high level of 24 h UTP and serum creatinine, low level of serum albumin, it may indicate a poor prognosis. Especially, increased 24 h UTP quantification is an independent risk factor for poor prognosis. The study has implications for doctors that LN children with high‐risk factor can be identified early by their baseline features and early response. If this happens in LN children, the high doses of cyclophosphamide was needed for them, the maximum dose was 750 mg/m^2^. If cyclophosphamide leads to secondary immunodeficiency or other side effects such as serious vomiting and severe hair loss, cyclosporine or tacrolimus, even rituximab, was needed. This study helps us to change our strategy and choose the right immunotherapy treatment for them.

## Author Contributions

W.Q.J. collected clinical data and drafted the article. H.F.J. collected and analyzed clinical data. Y.B. and W.C.Y. helped W.Q.J. to collect data. W.W., Z.L.Q., and D.Y.Q. provided assistance in data interpretation. S.H.M. and W.W. designed the topic and S.H.M. approved the final version of the manuscript.

## Ethics Statement

The studies involving human participants were reviewed and approved by the ethics committee, and all participants provided their written informed consent before their involvement.

## Consent

Written informed consent for publication was obtained from all participants.

## Conflicts of Interest

The authors declare no conflicts of interest.

## Data Availability

The authors have nothing to report.
